# Mr. Ring, on Inoculation

**Published:** 1805-11-01

**Authors:** John Ring

**Affiliations:** New Street, Hanover Square


### Mr. Ring, on Inoculation.

[ Continued from our last, pp. 355—361. ]

Dr. Aubert, in his "Report on Vaccine Inoculation," relates a case, which occurred in a woman who bad been previously inoculated for the small-pox by Mr. Wachsel, and in whom inoculation succeeded. This woman afterwards lost a child whom she suckled, by the confluent small-pox. Two or three days afterwards, nineteen pustules, perfectly similar to those of the small-pox, appeared on her face; particularly on the cheek, where the hand of the child had constantly rested. The face swelled, and was painful; and the pustules maturated, formed scabs, and left pits behind. Mr. Wachsel reminded me of this case; of which he was a witness. Dr. Aubert does not affirm that the patient had any fever; but it is impossible to conceive such symptoms as the foregoing can exist without it. He allows that cases of local small-pox are sometimes accompanied with fever; which he attributes to the inflammation of the arm. This, however, is only an hypothesis; and of course, liable to be an error.

I lately communicated the case of Mrs. Watts of Fulham; whom I had twice called to see, after her recovery; but I did not find her at home. I have since seen her; and she informs me, that her case was much more severe than I stated it to have been. She had about thirty pustules, scattered over different parts of the body; and was so ill that she was obliged to have medical advice. She was not able to leave her bed for four days; nor her room for a week.

On referring to the analysis of Dr. Aubert's publication, in my treatise on the cow-pox, I find the case before mentioned; and also the following remarks, which, unfortunately, are not unseasonable even now.—"Dr. Aubert observes, that it is necessary to make an accurate distinction between the small-pox, and that local infection which is sometimes occasioned by inoculating with variolous matter, those who have had either the small-pox or the cow-pox. Instances of such local affection have already been adduced, in the course of this work; I shall, nevertheless, record this additional proof of their existence.

"Some children who had undergone vaccination a year before, were exposed to the infection of the small-pox, in the Small-pox Hospital; and at the same time were inoculated with variolous matter in each arm. In one of them a pustule appeared on the inoculated part, bearing an exact resemblance to a small-pock; from which matter was taken, which produced the perfect disease. Dr. Aubert observes, that instances of a similar kind are common, where a local pustule is excited by variolous matter, in those who have had the small-pox; whether the matter is applied by chance or design. Desoteux, in his treatise on inoculation, has published an account of experiments of this sort; where all whom he inoculated from one of those local pustules, had the small-pox as completely as if they had been inoculated from one who had the general affection."

The treatise on inoculation here alluded to by Dr. Aubert, was written by Dr. Desoteux, in conjunction with Dr. Valentin, formerly his pupil; who lately sent it to me, according to his promise when in England. I shall therefore avail myself of the opportunity of transcribing the passage in question; for the sake of such of your readers, as have not the work in their possession.—"Citizen Roume Saint-Laurent, National Commissioner at St. Domingo, read a memoir on inoculation to the Society of Arts and Sciences at the Cape, in 1792; in which he reported, that he had taken matter on the tenth day, from a puncture which he had made on his hand, and which was swollen in consequence of the variolous matter there inserted; and, having inoculated several subjects at Grenada, they all received the infection of the small-pox, as completely from the matter of this local pustule, as if it had been furnished by the real small-pox. He also communicated this anecdote to the Society at Haerlem."

The following cases, which have come to my knowledge, since the publication of those which I lately communicated, will afford additional evidence, that those who have had the small-pox are not totally insusceptible of the disease. Mrs. Robinson, of Woburn, had the small-pox very severely; and was much marked by the disorder. Many years after, she nursed a young lady, who was a great favourite; in consequence of which she had upwards of a hundred pustules, and a considerable degree of constitutional indisposition. This account was communicated to me by her grandson, Mr. Robinson, No. 279, Holborn; who saw her when labouring under the second attack.

Mr. Wachsel informs me, that he saw a woman, who had had the small-pox before, with about three hundred pustules, in consequence of nursing her child in the disease.

Mrs. Briggs, of Layton in Essex, informs me, that she had had the small-pox by inoculation when ten years of age; from which a proper cicatrix remains. When nursing one of her children in the disorder, she had ten or twelve pustules; and was very ill.

Mrs. Downing, No. 44, Wardour Street, was inoculated for the small-pox when eleven years of age. Her arm rose, and a considerable number of persons were inoculated from her. She had a violent pain in her head and limbs; and was extremely ill. She had a very high fever. Her arm continued in a state of ulceration three months; and she has a very distinct scar remaining. Eight years ago, she lost a child by the small-pox. Four days after the death of the child she was seized with a violent sickness, fever, and pains in her head, back, and limbs. In about six or seven days, she had an eruption all over her body; and on one of her hands. I have seen several large and deep pits on her breast; which were left by this eruption.

An uncle of Mr. Drene, No. 8, Adams Mews, was inoculated for the small-pox. His arm rose, he sickened, and had an eruption; and the inoculator assured him he was safe; nevertheless, he has since had the small-pox.

Mrs. Miller, No. 6, Little-Argyle Street, had the small-pox when young. Nevertheless, about two years ago, when nursing an infant labouring under that disease, she had an eruption of several pustules in her face, and particularly about the eye; which, in consequence of considerable tumefaction of the lids, became perfectly closed. She declared to Mr. Hyde in Swallow Street, to whom she was a tenant, that she had such agonising pain in her head, as she had never suffered on any other occasion.

Two children vaccinated by me, have had slight attacks of the small-pox, since my last communication. One is the child of Mr. Lightfoot, No. 13, Lancashire Court, Bond Street. Mrs. Lightfoot recollects that a considerable quantity of matter was taken from this child, two days following. Many persons are of opinion, that this lessens the security. At any rate it is a duty, once more to recommend to every practitioner, as far as he possibly can, to try to preserve a supply of matter for himself, either by successive inoculations of the poor, or by means of vaccinators, on which, if repeatedly charged, it will keep good for many months. This will prevent the vesicles, in patients who are vaccinated gratuitously, from being too much exhausted.

The other is a child of Mr. Deacon, in Phillips's Gar-

 dens, dens, Tottenham-Court Road. I know of no peculiarity in this case. The appearance was regular: as a proof of which, the mother recollects, that an eminent inoculator vaccinated two or three children from her infant. I am now inclined to believe, that these, and some other well authenticated cases of a similar kind, are to be ascribed to the greater susceptibility of small-pox in some habits than in others; and not to any other disease with which the patient was affected, nor to the exhaustion of the arm. Could these causes operate so far, as to leave the subject still susceptible, I cannot but suspect the occurrence of the small-pox after vaccination would be more frequent; especially in public institutions, where the poor furnish a supply of matter for the rich. Nevertheless, from the fallacy of human judgement, I would seriously advise, that matter may always be taken with great caution; and no pustule be exhausted more than is necessary.

Dr. Nelson lately informed me of a decided case of the small pox, in a child who had been vaccinated at the Vaccine Pock Institution, in whom he had witnessed the regular progress of the cow-pock. The case occnrred in a child of Mr. M'Pherson, in Fitzroy Market; and I believe no one who saw it, could possibly doubt of its being the small-pox.

Dr. Nelson some time ago informed me of a case of the small-pox occurring a second time. It was communicated to him by Dr. Plowden, of Arundel. I therefore wrote to him on the subject; and he was so obliging as immediately to favour me with the following answer.

"*Arundel, September* 13, 1805.

"Sir,

"I yesterday received your letter, and shall be happy if any information I can give, concerning a person's twice having had the small-pox, should contribnte towards removing the doubts which exist, of the superiority of vaccination.

"In the year 1738, the small-pox committed its furious ravages in this place, and is said to have destroyed one out of every seven whom it attacked. Amongst the number of infected was William Birt, at that time about eighteen months old. It has been a tradition here, as long as the oldest of the inhabitants can remember, that this boy was supposed to have been dead; and that his body was purchased by Mr. Birch, a surgeon, for the purpose of dissection. He, however recovered; and for the rest rest of his days, was a living monument of the havoc the disorder had made. I knew him well; and do not recollect many instances of a person more fretted and seamed by the small-pox than he was.

"The marks he bore, were deemed a sufficient security against any future infection of the small-pox. He was therefore appointed to attend on variolous patients in the pesthouse; a kind of lazaretto at the skirts of the town.

"One woman died of the small-pox in the house, on the 15th of February, 1799, and another a few days after. William Birt, the subject of your inquiries, sickened on the 28th of the same month; and the eruption appeared on the fourth day. I saw him on the sixth day of the disease, being sent for by the family to give my opinion on the possibility of its being the small-pox; which none of them could believe, from the evident marks of his having had the complaint.

"On inquiry I found, that he had been exposed, a short time before, to variolous contagion; and that his disorder commenced with the symptoms usually attending the worst kind of the small-pox. The eruption was confluent; and his throat was severely affected. He was removed from his home, on the day I visited him, to the pest house; where he died on the twelfth day of the disease.

"If this detail can be of any service, I beg you will accept it, with my sincere wishes, that it may contribute towards the advancement of your pursuits.

"I am, &c.

"William Plowden."

The following is another instance of the small-pox occurring a second time. A child of Mr. King, No. 3, Printer Street, Blackfriars, was inoculated for the small-pox, at the Small-pox Hospital, in March, 1798. A pustule rose; the patient sickened at the usual time, and was very ill three days; after which an eruption took place, consisting of ten very large pustules, scattered over different parts.

Mrs. King went to the hospital three times a week, for the space of three weeks; and had several doses of physic for the child. On the last day of her attendance, she was assured by Mr. Wachsel, the child was safe, and she need not attend any more. Mr. King confirms this statement in all the essential particulars. He several times accompanied Mrs. King, when she went to the hospital; on account of her not being able to carry the child so far.

The day after they last attended at the hospital, the child again sickened, and had a violent vomiting. Two days afterwards, a very considerable eruption took place; which the neighbours supposed to be the measles. A few days after, Mr. Ridout was desired to attend the child; and being informed that she had been inoculated at the Small-pox Hospital, thought it necessary to send for Dr. Woodville. Dr. Woodville saw her in the course of the day. He told Mr. Ridout, it was a case of small-pox occurring a second time; and the second instance he had seen, in which there could be no doubt of the fact. Mr. Ridout informs me, that he advised Dr. Woodville to take notes of the case, as he was writing the history of inoculation; but is not certain whether he did so or not.

On the next day, Dr. Woodville informed Mr. Ridout, he had consulted the register, and found that this child, and fourteen others, were inoculated from a man in the hospital, who had the natural small-pox; and that all of them, except one, had taken the disease.

When Mr. King went to Dr. Woodville, and told him his child had the small-pox, the Doctor replied, he might as well tell him she had the plague. However, when he saw it, he was convinced. He continued to attend her, with Mr. Ridout, during the whole course of the disorder; which was about three weeks. The symptoms were very violent. Circular ulcerations took place, where some of the pustules had been formed in the original disease, and threatened mortification; but this was prevented by the internal use of bark, acids, and opium; and the external application of powder of bark.

Mrs. King declares, that on the sixteenth day, the child appeared to be in a dying state, and sometimes without any sign of life, for seven hours and a half. At length, however, she recovered; and she still bears indelible marks of the two attacks of this dreadful disease, which she has undergone.

Mr. Ridout favoured me with a statement of the foregoing case, as far as it fell under his observation; and Mr. Wachsel, who also attended the case, acknowledges that the second attack was the small-pox.

Two days ago, I was informed, that a child of Mr. Barrel, No. 2, Beaumont Place, vaccinated at the Small-pox Hospital, had the small-pox. I found her in good health; but was told she had been indisposed previously to the eruption; eruption; which was very slight, but appeared to me to be variolous. I have since seen an eminent practitioner, who entertains a different opinion; but is in the same predicament with me, not knowing how to put the matter to the test, consistent with his duty to those who put themselves under his care. It is worthy of notice, that in this, and almost every other instance, where I have inquired into a failure, or a supposed failure, in the inoculation of the cow-pock, I have heard of a failure, or a supposed failure, in the inoculation of the small-pox. Mrs. Barrel informs me, that her brother was inoculated for the small-pox, and declared safe; but about a year after, he had the natural disease.

A similar coincidence has lately happened in two children of Mr. Chitty, in East Street, Red Lion Square; one of whom was inoculated for the small-pox six years ago, the other for the cow-pock sixteen months ago, by Mr. Drewe of Gower Street. They have both lately had the small-pox, in a favourable way. This tends to confirm the opinion I gave in the former part of this paper, that the failures which now and then occur after inoculation, are owing to an idiosyncracy; of which those in Fullwood's Rents are a remarkable instance.

---

Dr. De Carro has transmitted to me a memoir, entitled, "Notice Bibliographique sur La Docteur Jenner," by Dr. Valentin, of Marseilles. This memoir is, in a great measure, a translation of the "Life of Dr. Jenner," in the "Public Characters;" which Dr. Valentin has thought proper to ascribe to me. It is of little importance to the public, who is the author of that article; but if any part of it is erroneous, it is of some importance that it should be corrected, while the circumstances are still recent; and many persons are living who can either attest, or refute them.

Dr. Valentin says, Dr. Jenner has been elected Mayor of Cheltenham. It is not Cheltenham, but Berkeley, his native place, which has done him, and themselves, that honour. He has also been presented with the freedom of the City of Dublin; and what is more flattering to him, as a medical character, a diploma from the University of Madrid. Dr. Valentin bears testimony to the discovery of Dr. Jenner, that it is the most important ever made.

He relates an anecdote, worth recording. When he was at Bath, about two years ago, Dr. Gibbes informed him, that when his father went to Oxford, forty years before Dr. Jenner's discovery, he asked his servant, on his arrival, whether he was not afraid of the small-pox, which at that time prevailed in the city; to which he replied, he should not catch it, because he had had the cow-pox; which was known in Wiltshire to be a security against that disease.

Dr. Waterhouse, as well as Dr. Valentin, has concluded his biographical sketch of Dr. Jenner, borrowed from the Public Characters, with the Latin and English verses, which are prefixed to that memoir in the original; beginning with *Te mater omnis, &c.* Dr. Waterhouse has led Dr. Valentin into an error, by attributing the Latin lines to Horace. They were prefixed by Dr. Best to his Thesis on Vaccination; and are taken from an Alcaic ode, signed *Ed. Hannes, ex Œde Christi Oxon:* addressed to Sydenham, and prefixed to his *Schedula Monitoria de Novæ Febris Ingressu.*

Dr. Waterhouse having subjoined the translation, Dr. Valentin ascribes to him what was written by me. The Latin and English are inaccurately copied in the French Memoir; I therefore beg leave to insert a correct copy here.

Te mater omnis, te lachrymabilis

Accuret uxor, ne caducum

Orba virum puerosque ploret:

Seu confluentes forte timet notas

Matura virgo, tu faciem eripis

Periclitantem, protegisque

Delicias juvenum futuras.

TRANSLATION.

To thee shall weeping wives and mothers fly,
Or see their husbands, and their children die;
To thee, the virgin trust her lovely face,
Or some rude blemish rifle ev'ry grace.

Oh, ward the perils that around her wait!
Oh, shield her beauties from impending fate!
Nor let a cruel pestilence destroy
The hope of youth, and pledge of future joy.

In a letter which accompanied Dr. Valentin's Memoir Dr. De Carro says, he has lately read in several newspapers, that Dr. Jenner is preparing a new work, in which he will prove, that the children of parents who have the cow-pock are insusceptible of the small-pox. This information, from whatever quarter it may have been derived, is erroneous. Dr. De Carro himself is so far from assenting to the proposition, that he declares he has had a number ber of proofs to the contrary. At present, however, he says, he meets with no proof of the kind, because the small-pox is utterly unknown among the people of Vienna. It is there completely exterminated; and neither attacks the children of those who have had the cow-pock, nor of those who have had the small-pox.

Dr. Anderson, Physician-General at Madras, has transmitted to me a tract, entitled, "Correspondence for the Extermination of Small-pox," together with some articles which he has inserted in the Government Gazette. From one of them, dated December 19, 1804, we learn, that by the last returns, 216,000 persons had been vaccinated in the settlement of Madras, and 26,000 in Ceylon. In order still further to promote the practice, he proposes to establish fixed and permanent institutions; so that every village may have the advantage of vaccine inoculation within itself.

He has added a letter, received from a Bramin in the neighbourhood; who informs him, he has perused his publications on the subject of the illustrious Jenner's immortal discovery; by which many of our fellow creatures in India, as well as in Europe, have been protected from that loathsome, painful, and fatal disease, the small-pox.

He declares, that he himself, and many others, have been eye witnesses to the mild and harmless nature of vaccination; and that it is therefore greatly to be wished, the natives of India may acquire an intimate knowledge of the practice, in order to preserve the lives of people of all conditions.

"On this account," says he, it might be useful to remove a prejudice in the minds of the people, arising from the term cow-pox being literally translated co-mary, in the advertisement which has been translated in our Tamul tongue; whereas there can be no doubt, *that it is a drop of nectar from the exuberant udders of the cows in England*; and no way similar to the humour discharged from the tongue and feet of diseased cattle in this country."

I am, &c.

*New Street, Hanover Square.* JOHN RING

